# NLRP3 regulates immunometabolism, impacting microglial function in Alzheimer’s disease

**DOI:** 10.1002/alz.095562

**Published:** 2025-01-09

**Authors:** Roisin M. McManus, Max P Komes, Angelika Griep, Francesco Santarelli, Stephanie Schwartz, Juan Perea, Michelle‐Amirah Khalil, Johannes C.M. Schlachetzki, David S. Bouvier, Mario Lauterbach, Susanne V. Schmidt, David Gomez‐Cabrero, Jesper Tegner, Christopher K. Glass, Karsten Hiller, Eicke Latz, Michael T. Heneka

**Affiliations:** ^1^ German Center for Neurodegenerative Diseases (DZNE), Bonn, NRW Germany; ^2^ Institute of Innate Immunity, Bonn, NRW Germany; ^3^ German Center for Neurodegenerative Diseases (DZNE), Bonn Germany; ^4^ University Hospital Bonn, Bonn Germany; ^5^ Centro de Biologia Molecular Severo Ochoa (CSIC‐UAM), Madrid Spain; ^6^ Technische Universität Braunschweig, Braunschweig Germany; ^7^ University of California, San Diego, San Diego, CA USA; ^8^ Luxembourg Centre for Systems Biomedicine, University of Luxembourg,, Belvaux Luxembourg; ^9^ Institute of Innate Immunity, Bonn Germany; ^10^ Institute of Innate Immunity, University Hospital Bonn, Bonn Germany; ^11^ Centro de Investigación Biomédédica, Pamplona Spain; ^12^ Karolinska Instituet, Stockholm Sweden; ^13^ Luxembourg Centre for Systems Biomedicine (LCSB), University of Luxembourg, Luxembourg Luxembourg

## Abstract

**Background:**

Our laboratory has demonstrated that the NLRP3 inflammasome has a critical role in the microglial innate immune response to Alzheimer’s disease (AD)‐related peptides, triggering the release of cleaved‐caspase‐1 and IL‐1β. NLRP3 activation was found in post‐mortem tissue from individuals with AD (Heneka et al., 2013) and in transgenic models of AD (APP/PS1 mice). However, APP/PS1 mice deficient for NLRP3 were protected from AD‐pathology and neuroinflammation. We wanted to examine the wider implications of NLRP3 deficiency and uncover other pathways and mechanisms employed by microglia to protect the brain from the buildup of Aβ.

**Method:**

Single cell RNA sequencing (scRNAseq) and ATAC sequencing was performed on CD11b^+^ cells from the brains of wildtype, APP/PS1, NLRP3^−/−^ and APP/PS1.NLRP3^−/−^ mice. Microglia were prepared from wildtype and NLRP3^−/−^ mice to assess metabolic function, phagocytosis and for flow cytometric analysis. Targets were investigated in the post‐mortem brain tissue of those with and without AD. In a separate experiment, APP/PS1 mice were treated with NLRP3‐targeted inhibitors.

**Result:**

ScRNAseq found a unique cluster of microglia in APP/PS1.NLRP3^−/−^ mice with pathways related to phagocytosis and glutamate metabolic signaling, with a specific increase in the glutamate transporter *Slc1a3*. Metabolically, NLRP3^−/−^ microglia had increased mitochondrial and metabolic function with altered levels of the metabolite α‐ketoglutarate. NLRP3^−/−^ microglia had greater Aβ phagocytosis than the wildtype cells, which was strictly associated with α‐ketoglutarate availability and epigenetic regulation. Importantly, we were able to replicate these findings in human cells and using NLRP3‐specific inhibitors *in vitro* and *in vivo*.

**Conclusion:**

We have identified a new mechanism where loss of NLRP3 influences glutamine/glutamate metabolism, which modulates α‐ketoglutarate to affect epigenetic regulation and gene transcription, boosting downstream phagocytic activity (McManus et al., in revision). This pathway is conserved between murine and human cells. Critically, we can mimic this effect *in vivo* using NLRP3‐specific pharmacological inhibition. Together, our data strengthens NLRP3 as a master‐immune regulator and an important target in the treatment of AD and dementia.

